# Supplementary material for: Determinants of neonatal near miss among neonates admitted to public hospitals in Southern Ethiopia, 2021: A case-control study
Source: PLoS One. 2022 May 6;17(5):e0268041. doi: 10.1371/journal.pone.0268041 (PMC9075625; doi:10.1371/journal.pone.0268041)
Supplement: S1 Data — (DOCX) [file pone.0268041.s001.docx]

## Data collection tool for a study titled “Determinants of Neonatal Near Miss among Neonates Admitted to Public Hospitals of Southern Ethiopia, 2021: A Case-Control Study

| **S No** | | **Questions** | | | | **Response** | | | | | | | | | | | **Skip** |
| --- | --- | --- | --- | --- | --- | --- | --- | --- | --- | --- | --- | --- | --- | --- | --- | --- | --- |
| **Part I: Identifications** | | | | | | | | | | | | | | | | |  |
| 101 | | Hospital Name | | | | | 1. NEMMH 2. Shone primary Hospital | | | | | | | | | |  |
| 102 | | Hospital code | | | | | **____/___/____** | | | | | | | | | |  |
| 103 | | Date form filled to start | | | | | ____/___/____ | | | | | | | | | |  |
| 104 | | Woreda /District | | | | | ________________________ | | | | | | | | | |  |
| 105 | | Kebele | | | | | ________________________ | | | | | | | | | |  |
| 106 | | Admission date | | | | | _____/____/____EC | | | | | | | | | |  |
| 107 | | Maternity record number/Medical Reg. Number | | | | | _________________ | | | | | | | | | |  |
| **Part II Socio demographic and economic characteristics of respondents** | | | | | | | | | | | | | | | | | **Skip** |
| 201 | How old are you? | | | 1. [___________]  2. I don’t know------------ | | | | | | | | | | | | |  |
| 202 | What is your marital status? | | | 1. Married | | | | | | 1. Divorced | | | | | | |  |
|  |  |  |  | 1. Unmarried/single | | | | | | 1. Widowed | | | | | | |  |
| 203 | What is your religion? | | | 1. Orthodox | | | | | | 1. Protestant | | | | | | |  |
|  |  |  |  | 1. Catholic | | | | | | 1. Muslim | | | | | | |  |
|  |  |  |  | 1. Other | | | | | |  | | | | | | |  |
| 204 | What is your ethnicity? | | | 1. Hadiya | | | | | | 1. Kembata | | | | | | |  |
|  |  |  |  | 1. Siltei | | | | | | 1. Guraghe | | | | | | |  |
|  |  |  |  | 1. Wolaita | | | | | | 1. Others | | | | | | |  |
| 205 | What is your educational status? | | | 1. No formal education | | | | | | 1. Primary(1-8^th)^ | | | | | | |  |
|  |  |  |  | 1. Secondary (9-12^th)^ | | | | | | 1. College and above | | | | | | |  |
| 206 | What is your occupation? | | | 1. House wife | | | | | | 1. Merchant | | | | | | |  |
|  |  |  |  | 1. Farmer | | | | | | 1. Daily laborer | | | | | | |  |
|  |  |  |  | 1. Government employer | | | | | | 1. Others | | | | | | |  |
| 207 | What is the educational level of your husband? | | | 1. No formal education | | | | | | 1. Primary (1-8^th)^ | | | | | | |  |
|  |  |  |  | 1. Secondary (9-12^th)^ | | | | | | 1. College and above | | | | | | |  |
| 208 | What is his present Occupation? | | | 1. Farmer | | | | | | 1. Merchant | | | | | | |  |
|  |  |  |  | 1. Government employer | | | | | | 1. Daily laborer | | | | | | |  |
|  |  |  |  | 1. Others | | | | | | 1. No work | | | | | | |  |
| 209 | How many members are there within the family?(family size) | | | [________________] | | | | | | | | | | | | |  |
| 210 | Where is your place of residence? | | | 1. Urban 2. Rural | | | | | | | | | | | | |  |
| 211 | **Wealth index measurement** | | | | | | | | | | | | Yes= 1 | | | No=0 |  |
| 1 | Does your household have: | | | 1. Electricity | | | | | | | | |  | | |  |  |
|  |  |  |  | 1. Radio | | | | | | | | |  | | |  |  |
|  |  |  |  | 1. Television | | | | | | | | |  | | |  |  |
|  |  |  |  | 1. mobile telephone | | | | | | | | |  | | |  |  |
|  |  |  |  | 1. table | | | | | | | | |  | | |  |  |
|  |  |  |  | 1. chair | | | | | | | | |  | | |  |  |
|  |  |  |  | 1. Bed | | | | | | | | |  | | |  |  |
| 2 | Does anyone of your household member have? | | | 1. Bicycle | | | | | | | | |  | | |  |  |
|  |  |  |  | 1. An animal-drawn cart | | | | | | | | |  | | |  |  |
|  |  |  |  | 1. Motorcycle | | | | | | | | |  | | |  |  |
|  |  |  |  | 1. Bajaj | | | | | | | | |  | | |  |  |
|  |  |  |  | 1. A car/truck? | | | | | | | | |  | | |  |  |
| 3 | Do you have private home? | | | | | | | | | | | |  | | |  |  |
| 4 | Main material of the roof (observe) | | | | | | | 1. Grass 2. Metal/corrugated iron | | | | | | | | |  |
| 5 | What is the main source of drinking water for your household? (circle or tick on the options) | | | | | | | 1. Water from spring/ river/ pond 2. Dug well 3. Pressure Piped 4. Tap water | | | | | | | | |  |
| 6 | What type of fuel does your household mainly use for Cooking?(code based on the respective number) | | | | | | | 1. Animal dung 2. Wood 3. Charcoal 4. Electricity | | | | | | | | |  |
| 7 | Does any member of this household have a bank or microfinance saving account | | | | | | | 1. No 2. Yes | | | | | | | | |  |
| 8 | How many of the following animals does the house hold have? (in number) | | | | | | | Milk Cows | | | | | |  | | |  |
|  |  |  |  |  |  |  |  | Ox | | | | | |  | | |  |
|  |  |  |  |  |  |  |  | Hen | | | | | |  | | |  |
|  |  |  |  |  |  |  |  | Goat/Sheep | | | | | |  | | |  |
|  |  |  |  |  |  |  |  | Donkey/Horse/Mule | | | | | |  | | |  |
| 9 | Does the household have cultivated agricultural land? | | | | | | | 1. No 2. Yes | | | | | | | | |  |
| 10 | If yes, in Hectares [__________________________] | | | | | | | | | | | | | | | |  |
| 11 | Did you rent/lease out land over the last 12 months? | | | | | | | | 1. No 1. Yes | | | | | | | |  |
| 12 | In the past 12 months how many quintals did you got? [list amount produced for each crop] | | | | | | | | 1. Teff | | | | | |  | |  |
|  |  |  |  |  |  |  |  |  | 2. Barely | | | | | |  | |  |
|  |  |  |  |  |  |  |  |  | 3. Wheat | | | | | |  | |  |
|  |  |  |  |  |  |  |  |  | 4. Maize | | | | | |  | |  |
| **PARTIII: OBSTETRIC CHARACTERISTICS OF RESPONDENTS** | | | | | | | | | | | | | | | | |  |
| 301 | | | Number of pregnancies (gravidity)? | |  | | | | | | | | | | | |  |
| 302 | | | Number of birth orders (parity)? | |  | | | | | | | | | | | |  |
| 303 | | | The duration between the current birth and the preceding birth in months? | | **/_______________/** | | | | | | | | | | | |  |
| 304 | | | Planning status of your last pregnancy while you got pregnant for the last time? | | 1. I had a plan and desire to that pregnancy | | | | | | | | | | | |  |
|  |  |  |  |  | 1. The pregnancy occurred earlier than desired) | | | | | | | | | | | |  |
|  |  |  |  |  | 1. The pregnancy occurred when no or more children were desired | | | | | | | | | | | |  |
| 305 | | | Did you have ANC visit while you were pregnant? | | 1. Yes  2. No | | | | | | | | | | | | **308** |
| 306 | | | How many times you had got the visit? | | [______________] | | | | | | | | | | | |  |
| 307 | | | Where was place of your last ANC visit? | | 1. Health center | | | | | | 2. hospital | | | | | |  |
|  |  |  |  |  | 3. Health post | | | | | | 4. other | | | | | |  |
| 308 | | | Where did you give birth before the current? | | 1. Health center | | | | | | 1. Hospital | | | | | |  |
|  |  |  |  |  | 1. Health post | | | | | | 1. Home | | | | | |  |
| 309 | | | In what Mode of delivery you got your child? | | 1. SVD | | | | | | 2. Instrumental deliver | | | | | |  |
|  |  |  |  |  | 3.C/S | | | | | |  | | | | | |  |
| 310 | | | Did you Have History of adverse birth outcome | | 1. Yes 2. No | | | | | | | | | | | | **401** |
| 311 | | | Types of adverse birth outcome | | 1. Spontaneous abortion | | | | | | | 1. Preterm birth | | | | |  |
|  |  |  |  |  | 1. Still birth | | | | | | | 1. Neonatal death | | | | |  |
|  |  |  |  |  | 5. Congenital malformations | | | | | | |  | | | | |  |

**PARTIV- MEDICAL CONDITIONS DURING PREGNANCY**

| 401 | Did faced Hypertensive disorders of pregnancies? | | 1. Yes 2. No | | | | | | 403 |
| --- | --- | --- | --- | --- | --- | --- | --- | --- | --- |
| 402 | What type of HDPs( record review) | | 1. Chronic hypertension | | | 2. Pre-Eclampsia | | |  |
|  |  |  | 2. Eclampsia | | | 4. Gestational hypertension | | |  |
| 403 | Did you sustained Gush of fluid before the onset of labor | | 1. Yes 2. No | | | | | |  |
| 404 | Did you sustained Vaginal bleeding before delivery | | 1. Yes 2. No | | | | | |  |
| 405 | Did you diagnosed with anemia during your pregnancy | | 1. Yes 2. No | | | | | |  |
| 406 | Did you diagnosed with DM during your pregnancy | | 1. Yes 2. No | | | | | |  |
| 407 | Did you diagnosed with malaria during your last pregnancy | | 1. Yes 2. No | | | | | |  |
| 408 | Did you diagnosed with syphilis during your last pregnancy | | 1. Yes 2. No | | | | | |  |
| 409 | Did you have sustained dystocia? | | 1. Yes  2. No | | | | | | **501** |
| 410 | If yes for the above question | | 1. Uterine rupture | | | | 1. Prolonged labor | |  |
|  |  |  | 3. Feto-pelvic disproportion | | | | | |  |
| **SECTION V : HEALTH SYSTEM RELATED FACTORS (ACCESS TO SKILLED MATERNITY CARE)** | | | | | | | | | **Skip** |
| 501 | | How long does it take you to walk to reach nearby health facility from your home? | | Minute/hours [_______]  I don’t know---------------- | | | | |  |
| 502 | | What means of transport did you use while you were going to health facility for the last time? | | 1. on foot | | | | 1. Vehicles |  |
|  |  |  |  | 1. Ambulance | | | | 4. Strature |  |
|  |  |  |  | 1. Others | | | |  |  |
| 503 | | Who will decide when you want to go to health facility for maternity services? | | 1. Myself 2. Me and my husband   3. My husband | | | | |  |
| 504 | | Did you stayed at maternity waiting room? | | 1. Yes  2.No | | | | | 506 |
| 505 | | How long you stayed there? | | 1.Less than one week  2. One week  3.More than one week | | | | |  |
| 506 | | Are you member of women health development army (WHDA)? | | 1. Yes 2.No | | | | |  |
| 507 | | Are you model hose hold (assure by observing certificate) | | 1.Yes  2. No | | | | |  |
| **SECTION VI: NEWBORN RELATED CHARACTERISTICS** | | | | | | | | |  |
| 601 | | Fetal presentation during delivery | | | 1. Cephalic  2. Breech  3. Transverse/face/brow  4. Others | | | |  |
| 602 | | Gestational age in weeks | | | [__________________] | | | |  |
| 603 | | Birth weight in gm. | | | [__________________] | | | |  |
| 604 | | Fetal malformation | | | 1. Yes 2. No | | | |  |
|  | |  | | |  | | | |  |

## Annex3: Abstraction checklist for Neonatal near miss cases

| **Part VI: Neonatal Near-Miss Criteria’s** | | | |
| --- | --- | --- | --- |
| **Pragmatic Markers** | | | |
| 601 | Gestational age | ______(in week) | |
| 602 | Birth weight | ______(g) | |
| 603 | Apgar score at 5th min | _______ | |
| **Management Severity Criteria’s** | | Yes =1 | No=0 |
| 604 | Use of intravenous antibiotics up to 7 days and before 28 days |  |  |
| 605 | Nasal CPAP |  |  |
| 606 | Any intubation |  |  |
| 607 | Use of phototherapy in the first 24 hour |  |  |
| 608 | Cardio pulmonary resuscitation |  |  |
| 609 | Use of any vasoactive drug |  |  |
| 610 | Use of anticonvulsants |  |  |
| 611 | Use of surfactant |  |  |
| 612 | Transfusion of blood derivatives |  |  |
| 613 | Use of corticosteroid for treatment of refractory hypoglycemia |  |  |
| 614 | Any surgical procedure |  |  |
| 615 | Use of antenatal steroid |  |  |
| 616 | Parenteral nutrition |  |  |
| 617 | Congenital malformation – ICD-10 |  |  |
| 618 | Admission to NICU |  |  |
| **Is the newborn considered near-miss?** | |  |  |

**THANK YOU!!!!!**
