# Supplementary material for: Determinants of neonatal near miss among neonates admitted to public hospitals in Southern Ethiopia, 2021: A case-control study
Source: PLoS One. 2022 May 6;17(5):e0268041. doi: 10.1371/journal.pone.0268041 (PMC9075625; doi:10.1371/journal.pone.0268041)
Supplement: Attachment — Submitted filename: NNM comments.docx [file pone.0268041.s003.docx]

**General comments**

The manuscript has been written in a detail form. As well, it’s great public health importance. However, correct major problems such as coherence and paragraph structuring, and improve grammar flow.

**Specific comments**

**Comment 1:**

Abstract (Methods)- why data were collected only using an interviewer-administered structured questionnaire? Why not semi-structured? As well, what is “data abstraction tool” mean in your manuscript? Please, use the proper term.

**Comment 2:**

Abstract (Results)- the first sentence is incomplete. What does the term “respectively” imply?

**Comment 3:**

Background- it’s well written. However, consider the following points.

1. In the first paragraph of the background the authors preferred to use the 2017 year and/or older data. However, these data are already outdated as it is almost 6 years back. Thus, update the references.
2. Start the background with the definition of NNM and overview of the major problems under inquiry.

**Comment 4:**

Methods and Materials- it’s written in detail. However, incorporate and reason out the following points.

1. Do you think that the reader(s) of your manuscript will access reference number 38 that you have cited? Any justification?
2. Despite study area has been written in detail, the authors did not specify which health facilities have been included in their investigation. Thus, write in detail about your study health settings.
3. Avoid future tense in the paragraph. E.g.: “Three controls will be chosen for each near-miss case on the same day as the near-miss event.”
4. Who ascertained cases/controls in your study? How did you manage variations among your case/control ascertainers as you included multi-center? Did you apply any statistical test to check for consistency among case/control ascertainers?
5. Sampling procedure- why three study area have been selected? Why not all areas have been included?
6. Amharic as a local language? What about other non-amharic speakers? Hadiya, kambata, silte ethnic groups.
7. Data analysis- avoid future tense used in describing about PCA.
8. Report p-value of model fitness test.
9. Which multivariable and why?
10. Ethical consideration- it is unclear what authors have described for age >18 and <18 years old. As your primary participants were neonates, what it the importance of assent? Any justification?

**Comment 5:**

Results- multivariable table. Remove the column of p-value and use asterisk to show the significant variables.

Comment 6:

Discussion-avoid direct interpretation of the ODDS RATIO. Because, you already interpreted them in the results section. Simply name the significant variables and continue with further explanation.
