# Supplementary material for: Determinants of neonatal near miss among neonates admitted to public hospitals in Southern Ethiopia, 2021: A case-control study
Source: PLoS One. 2022 May 6;17(5):e0268041. doi: 10.1371/journal.pone.0268041 (PMC9075625; doi:10.1371/journal.pone.0268041)
Supplement: Attachment — Submitted filename: Response to Reviewers.docx [file pone.0268041.s004.docx]

**AUTHORS’ RESPONSE TO ACADEMIC EDITOR AND REVIEWERS**

1. **AUTHOR’S RESPONSE TO EDITOR**

**Dear: Devendra Raj Singh (Academic Editor)**

We thank you for a thorough reading and constructive comments and suggestions of our manuscript and for the opportunity to revise and resubmit. We are pleased to submit the revised research article “**Determinants of Neonatal Near Miss among Neonates Admitted to Public Hospitals of Southern Ethiopia, 2021: A Case-Control Study**” for your consideration in the special collection of Plos One. On the following pages, you will find our response to the editor’s comments. On behalf of my co-authors, I thank you for your consideration of this resubmission. We appreciate your time and look forward to your response.

Sincerely,

Aklilu Habte(MPH)(corresponding author)

[**aklilihabte57@gmail.com**](mailto:aklilihabte57@gmail.com)

**AUTHORS’ RESPONSE TO ACADEMIC EDITOR**

PONE-D-21-29699

Determinants of Neonatal Near Miss among Neonates Admitted To Public Hospitals of Southern Ethiopia, 2021: A Case-Control Study

PLOS ONE

Dear Dr. Habte,

Thank you for submitting your manuscript to PLOS ONE. After careful consideration, we feel that it has merit but does not fully meet PLOS ONE’s publication criteria as it currently stands. Therefore, we invite you to submit a revised version of the manuscript that addresses the points raised during the review process.

Please include the following items when submitting your revised manuscript:

**Journal requirements:**

**Comment1:** Please ensure that your manuscript meets PLOS ONE's style requirements, including those for file naming. The PLOS ONE style templates can be found at

https://journals.plos.org/plosone/s/file?id=wjVg/PLOSOne_formatting_sample_main_body. Pdf and <https://journals.plos.org/> plosone/s/file?id=ba62/PLOSOne_formatting _sample_title_authors_affiliations.pdf.

**Response:** Thank you for reminding us to adhere to the journal's submission guidelines, and we have endeavored to submit the revised version of the manuscript strictly following the PLOS ONE guidelines.

2. Please include additional information regarding the survey or questionnaire used in the study and ensure that you have provided sufficient details that others could replicate the analyses. For instance, if you developed a questionnaire as part of this study and it is not under a copyright more restrictive than CC-BY, please include a copy, in both the original language and English, as Supporting Information.

**Response:** Thank you for your suggestion, and we have included the data collection tools (Questionnaire) in the “supporting information” section of the “Revised manuscript with track changes” as **“S1-data collection tool for NNM”**, Line 506, on Page21

__________________________________________________________________________________________________________END________________________________________

THANK YOU!!!

**AUTHORS’ RESPONSE TO REVIEWERS**

**DEAR: REVIEWER#1**

We thank you for a thorough reading and constructive comments and suggestions on our manuscript and for the opportunity to revise and resubmit. We are pleased to submit the improved research article **“Determinants of Neonatal Near Miss among Neonates Admitted to Public Hospitals of Southern Ethiopia, 2021: A Case-Control Study”** for your consideration in the special collection of Plos One. On the following pages, you will find our response to the reviewers’ comments. On behalf of my co-authors, I thank you for your consideration of this resubmission. We appreciate your time and look forward to your response independently as for reviewer 1, 2, 3 and 4 respectively.

Sincerely,

Aklilu H. (MPH)(corresponding author)

[**aklilihabte57@gmail.com**](mailto:aklilihabte57@gmail.com)

**AUTHORS’ RESPONSE TO REVIEWER**S

The manuscript must describe a technically sound piece of scientific research with data that supports the conclusions. Experiments must have been conducted rigorously, with appropriate controls, replication, and sample sizes. The conclusions must be drawn appropriately based on the data presented.

Reviewer #1: Yes

Reviewer #2: Yes

Reviewer #3: Yes

Reviewer #4: Yes

___________________________________________________________________________

**Response:** We thank the reviewer for the kind words regarding the soundness of our study.

2. Has the statistical analysis been performed appropriately and rigorously?

Reviewer #1: Yes

Reviewer #2: Yes

Reviewer #3: Yes

Reviewer #4: Yes

**Response:** We thank the reviewer for the kind words regarding the appropriateness and rigorousness of our work.

3. Have the authors made all data underlying the findings in their manuscript fully available?

The PLOS Data policy requires authors to make all data underlying the findings described in their manuscript fully available without restriction, with rare exceptions (please refer to the Data Availability Statement in the manuscript PDF file). The data should be provided as part of the manuscript or its supporting information, or deposited to a public repository. For example, in addition to summary statistics, the data points behind means, medians and variance measures should be available. If there are restrictions on publicly sharing data—e.g. participant privacy or use of data from a third party—those must be specified.

Reviewer #1: Yes

Reviewer #2: Yes

Reviewer #3: Yes

Reviewer #4: Yes

**Response:** We thank the reviewer for assuring the availability of all data underlying the findings in their manuscript.

4. Is the manuscript presented in an intelligible fashion and written in standard English?

PLOS ONE does not copyedit accepted manuscripts, so the language in submitted articles must be clear, correct, and unambiguous. Any typographical or grammatical errors should be corrected at revision, so please note any specific errors here.

Reviewer #1: Yes

Reviewer #2: Yes

Reviewer #3: No

Reviewer #4: No

**Response:** We appreciate the reviewer's remarks on the way the information in the paper was represented. While dealing with individual comments, we attempted to address the comments and suggestions raised by reviewers 3 and 4.

**AUTHORS’ RESPONSE TO REVIEWER**#**1**

**Comment1:** regarding Sample size calculation…..Why did you take the percentage of cases exposed to old maternal age …….? What is the linkage between your variables

**Response:** Thank you very much for taking the time to review our work and for your positive feedback. To determine the sample size, we employed a double population proportion formula. To get the largest sample size possible, we used various covariates (maternal age, receiving less than 4ANC visits, and instrumental delivery) across multiple studies conducted in different countries. The largest sample size was obtained using the variable old maternal age (Age>35 years) among the aforementioned factors. was discovered.

In terms of the selected variable's relation to NNM, older maternal age has been found as a significant determinant of NNM across several studies.

For more clarity, we have added a table(Table1) that shows the way we followed while we were computing the sample size and is incorporated and highlighted in the “Sample size determination” section of the “Revised manuscript with track changes”, Line174-175, Page6

**Comment2:** Ethical consideration and consent to participate…. Do you think issuing unique ID numbers ensure confidentiality?

**Response:** Thank you for your inquiry. One approach for dealing with the issue of confidentiality during data gathering is to establish a unique ID number (Code). Rather than writing the respondents' names, it is preferable to assign a unique code, and that is why we use it in conjunction with other mechanisms to ensure confidentiality.

**Comment3:** SOCIODEMOGRAPHIC…(TABLE 1), Age of the mother ….calculate x2

**Response:** we have imputed it in the Tabe2 of “Revised manuscript with track changes”, Page11.

**Comment4:** Characteristics of the newborns Line 4 ……….The majority of cases (80.2%) and controls (314.5%) had a vertex presentation during…. Needs correction.

**Response:** Thank you for the meticulous review of the manuscript. We have corrected it and highlighted it in the “Characteristics of the newborns” section of the “Revised manuscript with track changes”, Line323, Page12.

**Comment5:** How do you measure birth preparedness and complication readiness (BPCR)? To classify the variable as good and poor?

**Response:** thank you for asking. In the "Definition and operationalization of variables" section of the initial manuscript, we defined the variable, birth preparedness and complication readiness (BPCR). It was assessed using eight WHO-recommended elements, with a good BPCR plan being determined as the woman implementing at least five of the eight items. For clarity, again we highlighted it in the “Definition and operationalization of variables” section of the “Revised manuscript with track changes”, Line 248-252, on Page8.

**Comment6:** Why you did not analyze AOR variables maternal age, family size, sex of the newborn, and Having hypertension during the last pregnancy

Response: Thank you for raising the pertinent question. While we were conducting the chi-square test, we didn’t get any significant variation in those mentioned variables across cases and control. For clarity: maternal age (χ2=1.692, p=0.429), family size (χ2=0.620, P =0.431),sex of the newborn (χ2=1.620, P=0.231), and Having hypertension during the last pregnancy (χ2=1.076, p=0.300).

__________________________________________________________________________________________________________END________________________________________

THANK YOU!!!

**AUTHORS’ RESPONSE TO REVIEWER**#**2**

**General Comment:** This is an interesting paper that clearly indicates the major public health importance Neonatal near miss in the low income country particularly Ethiopia. The paper is worth for publication after correcting the minor comments.

**Response:** Thank you very much for taking the time to review our work and for your positive feedback. We received your thoughtful review, along with helpful feedback and suggestions, as a valuable contribution to our ongoing work. We've attempted to respond to your comments and suggestions in the following manner.

**Commnet1:** There are several grammatical problems throughout the document, which require extensive English language editing. Professional English editing is needed

**Response:** We appreciate your meticulous review of the manuscript. We attempted to communicate with higher academicians in our university's department of foreign language and literature to address the grammatical faults, and we hope that those concerns have been addressed in the revised manuscript.

**Commnet2**: In the abstract part the authors used introduction, I believe it better to replace the word introduction with background.

**Response:** We have corrected and highlighted it in the “Abstract” section of the “Revised manuscript with track changes”, Line 35, on Page2

**Comment3:** In the abstract part the authors describe the conclusion and recommendation as Stakeholders at the zonal and regional levels need to step up their efforts to address the barriers that prevent health facilities from providing adequate and appropriate care. Furthermore, to prevent major neonatal problems, women who have not had an ANC and who deliver by Cesarean section require closer attention from their family and health care providers. This is generally unclear for the reader. Better to make the a bit specific the conclusion and recommendation in line with your findings.

**Response:** Thank you for your thoughtful comment, which we found to be very helpful in improving the readability of our work. We attempted to align the conclusion and recommendation sections with the findings of the study. The amendments were highlighted in the “Abstract” section of the “Revised manuscript with track changes”, Line 63-69, on Page2

**Commnent4:** In the method part your sampling procedures is very short. would you describe the sampling procedure?

**Response:** thank you for the comment and we have tried to illustrate the sampling procedure diagrammatically as Figure1 in the “Revised manuscript with track changes”, Line192-193, Page7

**Comment5:** I was wondering if you more explain The pragmatic and management criteria?

**Response:** Thank you for bringing up this idea. We tried to explain the two criteria in-depth in the method part of the original manuscript because they were the most important criterion for case ascertainment. For more clarity, we have highlighted in the “The population of the study” section of the manuscript, Line153-160, Page 5. In addition, they were ascertained in the “Definition and operationalization of variables” section, Line225-233.

**Comment6:** In the method section, data collection tools, could you describe more about your measurement instrument validity.

Response: We appreciate your advice and suggestion, which will help us improve the data quality. As a result of your recommendation, we've included those efforts that we've made to ensure the reliability and validity of the tools. The statements were highlighted in the “data quality management” section of the “Revised manuscript with track changes”, Line212-216, on Page7

**Comment7:** In the method part Sampling procedures for the selection of cases you have used consecutive sampling and for the selection of controls systematic sampling is used. what do you think about the generalization?

**Response:** We appreciate your insightful comments regarding the sampling procedure that we had been followed. The majority of researchers advised that the occurrence of a case is random and that using random sampling approaches would waste time for researchers. Due to the nature of the study, cases were more difficult to access than controls, thus we decided that using consecutive sampling for cases was a more viable option to save time. The majority of case-control studies used this method for case selection.

**Comment8:** in the data analysis technique you have deal with The Hosmer and Lemeshow goodness of fit test was used to assess the model's fitness. The variance inflation factor (VIF) was used to check for multicollinearity among independent variables. You should have to put the value obtained from the data analysis output with the respective interpretation.

**Response:** thank you for your positive comment and suggestion. As per your suggestion, we have added the aforementioned values from the model output and highlighted them in the “Data analysis” section of “Revised manuscript with track changes”, Line293-296, on Page9

__________________________________________________________________________________________________________END________________________________________

THANK YOU!!!

**AUTHORS’ RESPONSE TO REVIEWER**#**3**

**General comment:** Dear authors thank you very much for writing this very interesting topic. Neonatal mortality in Ethiopia is unacceptably high. These identifying determinant factors of neonatal near miss will be helpful for policy makers and stakeholders. Saying this I have listed my comments below.

**Response:** Thank you very much for taking the time to review our work and for your positive feedback. We received your thoughtful review, along with helpful feedback and suggestions, as a valuable contribution to our ongoing work. We've attempted to respond to your comments and suggestions as follow.

**Comment1:** In the Introduction section you define Neonatal near-miss (NNM) between the ages of 0 and 27 days. What about 28 days, in which group of age we called neonates?

**Response:** Thank you for your thorough review; as per your recommendation, we will correct it and highlight it in the "Abstract" section of the “Revised manuscript with track changes”, Line37, on Page2

**Comment2:** Rather than saying southern Ethiopia, it is better to write the specific area

**Response:** as per your suggestion, we have corrected and highlighted in the "Abstract" section of the “Revised manuscript with track changes”, Line42, Page2

**Comment3:** Why did you select an unmatched case-control study, why not matched case-control study?

**Response:** thank you for asking. we were conducting this study among consecutively selected neonates whose age groups range between 0 and 28 days and the possibility of obtaining confounders is less likely. In addition, currently published studies suggest that Matching in the design does not control for confounding by the matching factors, especially for those studies conducted in a short period of time, in days and weeks. Even it can introduce confounding by the matching factors even when they did not exist in the source population. In such cases, the matching process makes the controls more similar to the cases not only for the matching factor but also for the exposure itself. For the reasons mentioned above, we preferred to use unmatch case-control study. If you have any suggestions for improving our understanding of the importance of matching for this study, we would be grateful to hear.

**Comment4:** What is third maternal delay, be specific which delay?

**Response:** third maternal delay is one of the three delays that lead to maternal and child death. It was the interval of time between reaching the health facility and accessing the services needed. It took more than 1 hour to receive a delivery service deemed delayed and less than an hour deemed no delay. It was highlighted in the “Definition and operationalization of variables” section of the “Revised manuscript with track changes”, Line266-268, on Page9

**Comment5:** How do you measure poor birth preparedness and complication readiness?

**Response:** thank you for asking. In the "Definition and operationalization of variables" section of the initial manuscript, we defined the variable, birth preparedness and complication readiness (BPCR). It was assessed using eight WHO-recommended elements, with a good BPCR plan being determined as the woman implementing at least five of the eight items. For clarity, again we highlighted it in the “Revised manuscript with track changes”, Line 247-251, on Page8.

**Comment6:** Your finding and your conclusion are very far apart. For instance have studied barriers of health facilities? Are studied health care workers at community level, you have listed all in the recommendation. I think it needs rewritten.

**Response:** Thank you for your thoughtful comment, which we found to be very helpful in improving the readability of our work. We attempted to align the conclusion and recommendation sections with the study's findings. The amendments were highlighted in the “Abstract” section of the “Revised manuscript with track changes”, Line 63-69, on Page2

**Comment7:** the background section needs synthesis and chronologic order

**Response:** We appreciate your thorough review of the manuscript, and we regard your idea being an important contribution to improving the readability of our work. We re-synthesized those statements and tried to retain their chronological order for a better idea flow, as per your suggestion. Those amendments were highlighted throughout the “Background” section of the “Revised manuscript with track changes”

**Comment8:** Several studies were conducted regarding neonatal near miss, however you were not addressed their limitation and the need of your study.

**Response:** thank you for the comment and we have tried to incorporate the statements and highlighted them in the “Background” section of the “Revised manuscript with track changes”, Line 129-133, Page 4.

**Comment9:** Very critical why select a study conducted in Brazil to calculate your sample size; there were many of study in Ethiopia?

Response: Thank you very much for taking the time to review our work and for your positive feedback. To get the largest sample size possible, we used various covariates (maternal age, receiving less than 4ANC visits, and instrumental delivery) across multiple studies conducted in different countries including Ethiopia. The largest sample size was obtained using the variable old maternal age (Age>35 years) among the aforementioned factors from a study conducted in Brazil. was discovered. Of the selected variable, older maternal age has been found as a significant determinant of NNM which gave the maximum sample size.

For more clarity, we have added a table (Table1) that shows the way we followed while we were computing the sample size and is incorporated and highlighted in the “Sample size determination” section of the “Revised manuscript with track changes”, Line174-175, Page6

**Comment10:** Discussion Well written

**Response:**Thank you for your positive feedback.

__________________________________________________________________________________________________________END________________________________________

THANK YOU!!!

**AUTHORS’ RESPONSE TO REVIEWER**#**4**

**General comment:** The manuscript has been written in a detail form. As well, it’s great public health importance. However, correct major problems such as coherence and paragraph structuring, and improve grammar flow. Overall, incorporate the specific comments forwarded in the word document.

**Response:** Thank you very much for taking the time to review our work and for your positive feedback. We received your thoughtful review, along with helpful feedback and suggestions, as a valuable contribution to our ongoing work. We've attempted to respond to your comments and suggestions as follow.

**Comment 1a:** Abstract (Methods)- why data were collected only using an interviewer-administered structured questionnaire? Why not semi-structured?

**Response:** thank you for asking. Regarding to the type of data collection tool, we used the interviewer-administered structured questionnaire. Because a structured questionnaire is a tool in which the interviewer asks a particular set of predefined questions. Questions are planned and created in advance, which means that all respondents are asked the same questions in the same order. They are used in large interview programs (anything over 30 interviews and more likely over 200 interviews in number) and may be carried out face-to-face, self-completion, and over the telephone, or depending on the respondent type, the content of the questionnaire and the budget.

On the other hand, A semi-structured questionnaire is a tool in which the interviewer asks only a few predefined questions while the rest of the questions are not planned. So, in the current study, we didn't see the importance of using a semi-structured questionnaire because there were no items that allowed the respondent to add his or her opinion on any intriguing subjects, which is why we didn't report it.

**Comment1b:** As well, what is “data abstraction tool” mean in your manuscript? Please, use the proper term.

**Response:** It was simply referred to as a "data extraction checklist," which was used to extract those parameters from medical records during case ascertainment and also to collect some variables from the maternal side. In addition, employing those criteria, some maternal and neonatal characteristics were extracted from records. the term is “data abstraction tool” was replaced by “data extraction checklist” and is highlighted in the “Abstract” section of the “Revised manuscript with track changes”, Line 49, Page 2.

**Comment2:** Abstract (Results)- the first sentence is incomplete. What does the term “respectively” imply?

**Response:** thank you for asking. The two criteria, pragmatic and management criteria, were used to identify NNM cases, and we reported the cases based on the criteria. But to make more clear, we have tried to make the statement more clear and it was highlighted in the“Abstract” section of the “Revised manuscript with track changes”, Line54-55, Page 2.

**Comment3:** In the first paragraph of the background the authors preferred to use the 2017 year and/or older data. However, these data are already outdated as it is almost 6 years back. Thus, update the references.

**Response:** Thanks for putting up this idea which is crucial to keep us with more updated references. Although the report covers neonatal mortality from 1990 to 2017, the reference is the most up-to-date(2019) source for information on the global burden of neonatal mortality.

**Comment4:** Do you think that the reader(s) of your manuscript will access reference number 38 that you have cited? Any justification?

**Response:** thank you for asking. We couldn't find the document on the internet, of course. When we went to the zonal health department office to inquire about the zone's population profile, they provided us with a hard copy of the document, which is why we reported it. We've removed it since it can confuse readers, and we've replaced it with a declaration that it's a zonal report. This was highlighted in the “Study area, Period, and design” section of the “Revised manuscript with track changes”, Line144, Page5.

**Comment5:**	Despite study area has been written in detail, the authors did not specify which health facilities have been included in their investigation. Thus, write in detail about your study health settings.

**Response:** thank you for the comment and we have tried to describe those hospitals involved in the current study in the “sampling procedure” section of the “Revised manuscript with track changes”, Line184-186, Page6. In addition, the sampling procedure was supplemented by a diagrammatic presentation as “Figure1”

**Comment6:** Avoid future tense in the paragraph. E.g.: “Three controls will be chosen for each near-miss case on the same day as the near-miss event.”

**Response:** was corrected and highlighted in the “The population of the study” section of the “Revised manuscript with track changes”, Line163, Page6.

**Comment7:** Who ascertained cases/controls in your study? How did you manage variations among your case/control ascertainers as you included multi-center? Did you apply any statistical test to check for consistency among case/control ascertainers?

**Response:** Thank you for taking the time to read our paper and for requesting us to explain how case and control ascertainers operate. To minimize the possible misclassification bias, cases and controls were ascertained based on the initial diagnosis made by higher experts like pediatricians, neonatologists, gynecologists, or residents in maternal and child health specialties. We have added and highlighted this in the “Definition and operationalization of variables” section of the “Revised manuscript with track changes”, Line231-233, Page8. Finally, we mentioned the possible misclassification bias as a limitation of our study in the “Revised manuscript with track changes”, Line468-469, Page21.

**Commenta8:** Sampling procedure- why three study area have been selected? Why not all areas have been included?

**Response:** In the selected study area (Hadiya Zone), there were a total of four hospitals, three of which (75%) were included in the current study. The reason for this is a problem with resource feasibility and time constraints. Fortunately, all of the hospitals selected had a high caseload enough to achieve the desired sample size within the study period.

**Comment9:** Amharic as a local language? What about other non-amharic speakers? Hadiya, kambata, silte ethnic groups.

**Response:** Thank you for your inquiry and which is crucial for data quality management. All of the data collectors assigned were bilingual in Amharic and the local language, therefore there were no linguistic difficulties to overcome during the data collection. This was highlighted in the “Data Collection Tools, Methods, and Personnel” section of the “Revised manuscript with track changes”, Line203-205, Page7. In addition, we used a two-pronged approach for the data collection: an interview and a document review, which helped us deal with the issues that arose as a result of language barriers.

**Comment10:** Data analysis- avoid future tense used in describing about PCA.

**Response:** We appreciate your meticulous review of the manuscript by giving your precious time. We have corrected and highlighted it in the “Data analysis” section of the “Revised manuscript with track changes”, Line276, Page9.

**Comment11:** Report p-value of model fitness test.

**Response:** we have reported and highlighted the “Data analysis” section of the “Revised manuscript with track changes”, Line292-293, Page10.

**Comment12:** Which multivariable and why?

**Response:** It referred to a multivariable logistic regression model that we used to find the significant determinants of NNM after adjusting for potential confounders.

**Comment13:** Ethical consideration-it is unclear what authors have described for ages>18 and <18 years old. As your primary participants were neonates, what it the importance of assent? Any justification?

**Response:** thank you for asking. Just the majority of the data were collected from the maternal side. There were 3 respondents whose age was below 18 years and the assent was taken for them

**Commennt14:** Remove the column of p-value and use the asterisk to show the significant variables.

**Response:** We removed that specific column from table 6 as per your suggestion.

Comment15: Discussion-avoid direct interpretation of the ODDS RATIO. Because you already interpreted them in the results section. Simply name the significant variables and continue with further explanation.

**Response:** Thank you for your comment which enables us to avoid redundant ideas. As per your suggestion, we have removed those redundant statements and highlighted them throughout the “Discussion” section of the “Revised manuscript with track changes”.

__________________________________________________________________________________________________________END________________________________________

THANK YOU!!!
